# Simulation Based Education for Building Skills in the Management of Emotional Dysregulation and Self-Harm on Children and Adolescent Mental Health Inpatient Wards

**DOI:** 10.1192/bjo.2025.10299

**Published:** 2025-06-20

**Authors:** Antonia Winney, Damir Rafi, Naomi Tomlinson, Sam Ter-Horst

**Affiliations:** Maudsley Learning, London, United Kingdom

## Abstract

**Aims:** Professionals in Child and Adolescent Mental Health Services (CAMHS) inpatient settings frequently manage cases of heightened emotional dysregulation, self-harm, and risky behaviours. Successful management required specialised techniques and a deep understanding of both therapeutic interventions and legal frameworks.

We have created a one-day simulation course run multiple times reaching approximately 80 members of staff working across different Child and Adolescent Mental Health Services (CAMHS) units. The deliveries for this run from November 2024 through to March 2025, and are therefore still in progress at the time of submission. The course focuses on the use of Dialectical Behaviour Therapy (DBT) techniques and crisis de-escalation strategies. It also highlights the importance of legal frameworks, family involvement and inter-professional collaboration in the management and support of high-risk self-harm or suicidality.

**Methods:** Each course opens with introductions and an interactive icebreaker. The Maudsley Learning team, prioritises creating a psychologically safe environment that encourages learning and participation. To achieve this, we use a range of approaches, including a thorough icebreaker to build rapport, introducing the concept of the Basic Assumption, and integrating Equality, Diversity, and Inclusion (EDI) as a core focus throughout the day. We also emphasise learning through imperfect practice, fostering a supportive atmosphere where participants feel comfortable engaging and refining their skills. The course involves a full day of face-to-face teaching, with participants encountering five simulated patient scenarios. Each participant has the opportunity to engage in a realistic, high-fidelity patient scenario designed to replicate the challenges of working with children and young people in inpatient settings. Scenarios are tailored to focus on key issues such as crisis management, self-harm, and family dynamics. These simulations are followed by in-depth debriefing sessions, led by experienced faculty members, where participants will reflect on their use of techniques, risk assessment, and the legal and ethical considerations involved in each case.

**Results:** At the time of submission, not all deliveries of the course have taken place. However, initial feedback shows increased confidence across all the learning objectives and very positive written feedback. These will be presented in full in the poster.

**Conclusion:** This simulation course provides novel training to CAMH’s inpatient staff in DBT and de-escalation skills. It also provides a reflective space for staff to discuss the emotional impact of these presentations, and may be beneficial in reducing compassion fatigue.

